# Defining the necessity of the lipoic acid synthase *lias-1* in *Caenorhabditis elegans*

**DOI:** 10.17912/micropub.biology.001804

**Published:** 2025-10-15

**Authors:** Linnea B. Parker, Philippa Murray, Peter A. Kropp

**Affiliations:** 1 Biology, Kenyon College, Gambier, Ohio, United States; 2 National Institute of Diabetes and Digestive and Kidney Diseases, Bethesda, Maryland, United States; 3 Department of Orthopaedics, University of Maryland School of Medicine, Baltimore, Maryland, United States

## Abstract

Lipoic acid is an essential cofactor for multiple enzymes involved in aerobic respiration, and disruption to any of the pathways contributing to lipoic acid synthesis result in severe respiratory dysfunction. Despite the importance of lipoic acid, few studies have directly investigated the necessity of the lipoic acid synthase itself in eukaryotes. We have used *Caenorhabditis elegans* to address this gap and created CRISPR knockouts of *lias-1* , the first *lias-1* knockouts reported in *C. elegans* . These mutants show developmental arrest, sterility, and shortened lifespan which cannot be rescued by supplementing with exogenous lipoic acid suggesting a necessity for *de novo* lipoic acid synthesis in *C. elegans* .

**Figure 1. Impaired development of lias-1 mutant C. elegans:**
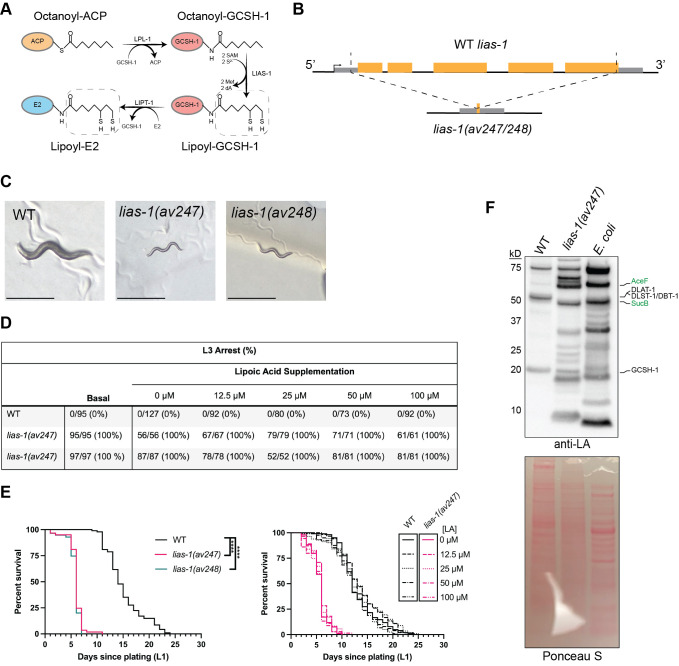
A) Schematic of the eukaryotic lipoyl relay pathway. *C. elegans* protein names are utilized in labels and the octanoyl/lipoyl moieties are indicated with labels with lipoic acid outlined in the dotted line. ACP: acyl carrier protein; E2: E2 subunit of oxoacid dehydrogenase complex. B) Schematic of the *lias-1* gene. Untranslated regions shown in gray; exons shown in orange. CRISPR cut sites and resulting deletion alleles shown with dotted lines. C) Representative images of WT and *lias-1* mutant animals 72 hours post egglay. Scale bar: 500 µm. D) Penetrance of L3 arrest phenotype in *lias-1* mutants. The individual number of animals assayed are shown for each condition as well as the percent (%) of animals arresting at L3 shown in parentheses. E) Kaplan-Meier survival curves of WT and *lias-1* mutant animals without (left) or with (right) supplemented LA. ****: p<0.0001 by log-rank analysis with Bonferroni correction for multiple comparisons. For LA supplementation, there were no significant differences due to LA treatment within the WT or *lias-1 (av247)* groups. n=57-94 animals across two independent trials for the untreated lifespan. n=71-122 animals across two independent trials for the LA-supplemented lifespan. Data from independent trials were aggregated into a single dataset for clarity. F) Representative western blot analysis of lipoylated proteins in *C. elegans* and *E. coli* samples. Molecular weights indicated on the left in kD. Prospective protein identities indicated on the right with *C. elegans* proteins in black and *E. coli* proteins in green. Ponceau S staining included to show protein loading. Lane order matches that of the blot, above.

## Description

Lipoic acid (LA) is a small thiol-containing cofactor that is essential for the function of the five oxoacid dehydrogenases which include pyruvate dehydrogenase and alpha-ketoglutarate dehydrogenase (Cronan, 2016, 2024). In eukaryotes, LA is synthesized *de novo* by a process known as the lipoyl relay in which octanoic acid is converted to LA through the insertion of two thiols by the lipoic acid synthase (LIAS) ( Figure 1A )(Cronan, 2024). This synthesis pathway is extremely well conserved, and loss of LA synthesis, either through direct loss of lipoyl relay or the upstream reactants, is associated with over a dozen mitochondrial diseases (Mayr et al., 2014; Tort et al., 2016; Cronan, 2020; Kastaniotis et al., 2020). Despite the importance of this pathway, there is a lack of research on the necessity of LIAS in multicellular organisms. To address this gap, we have generated novel and independently-generated CRISPR/Cas9 knockout strains of the *Caenorhabditis (C.) elegans* *LIAS* ortholog, *lias-1* , in which the majority of the coding sequence of *lias-1* has been deleted ( Figure 1B ). Deletion was confirmed by PCR and Sanger sequencing. Using *C. elegans* parlance, we have named these alleles *lias-1 (av247)* and *(av248)* .

We determined that both *lias-1* mutants arrested at the L3 larval stage ( Figure 1C, D) and had significantly shorter lifespans than WT animals ( Figure 1E ). Although mild mitochondrial or metabolic stress frequently have lifespan extending effects in *C. elegans* , severe mitochondrial stress does shorten lifespan or lead to embryonic lethality. This fact is particularly true for mutants in iron-sulfur cluster biogenesis (Ast et al., 2019; Kropp et al., 2021) which is necessary to provide the sulfurs for thiol insertion in lipoic acid (McCarthy and Booker, 2017; McCarthy et al., 2019; Warui et al., 2022). Therefore, these *lias-1* mutants are consistent with severe mitochondrial stress leading to decreased fitness.

As the two *lias-1* alleles were phenotypically identical, we used the *lias-1 (av247)* allele for further analyses. Some bacteria and fungi are capable of scavenging LA from the environment (Cronan, 2024), and *C. elegans* have been shown to be able to scavenge metabolic cofactors from their *E. coli* food source (Warnhoff and Ruvkun, 2019; Warnhoff et al., 2021), so we tested if supplementation with increasing concentrations of LA up to 100 mM could rescue the L3 arrest and lifespan of *lias-1 (av247)* animals. However, LA supplementation had no effect on the development or lifespan of the mutant or WT animals ( Figure 1D, E) indicating that exogenous LA is likely not bioavailable to *C. elegans* . This finding is in agreement with other studies demonstrating that exogenous LA provides a mild antioxidant effect, but cannot be incorporated into the oxoacid dehydrogenases (Feng et al., 2009; Mayr et al., 2011; Lavatelli et al., 2020; Bick et al., 2024; Pradel et al., 2024), although we did not test this incorporation directly in the LA-supplemented samples.

To confirm the abolishment of LA production in the *lias-1* mutants, we completed western blot analysis for lipoylated proteins. The canonical doublet of lipoylated DLAT-1 and DLST-1 / DBT-1 is absent in *lias-1 (av247)* . Surprisingly, we could still detect other lipoylated proteins in the *lias-1 (av247)* , but comparison of these bands with the lipoylated proteins of the bacterial food source ( *E. coli* OP50 ) showed that nearly all of the residual bands are the same size as *E. coli* proteins ( Figure 1F ). This result suggested that the *lias-1* mutants did not clear their gut of *E. coli* OP50 despite repeated washing in M9 and a 30-minute incubation which is normally sufficient for *C. elegans* to pass any residual bacteria. Decreased pharyngeal pumping and/or reduced defecation likely explain this phenotype, however, neither phenotype was assessed in this study. Intriguingly, some of the lipoylated protein bands in the *lias-1 (av247)* samples do not correlate with *E. coli* proteins ( *e.g.* at ~8 and ~70 kD). These bands may be bacterial proteins scavenged by *C. elegans* and modified, metabolized, or degraded to yield these novel bands, although we have no direct evidence for this possibility. More thorough analyses of these possibilities such as feeding *lias-1 (av247)* an LA auxotroph bacteria such as the *lipA-lplA* mutant TM131 will be necessary to make such determinations.

Although the LA synthesis pathway has been previously defined in *C. elegans* (Lavatelli et al., 2020), this is the first study to use genetic manipulation of *lias-1* rather than RNAi knockdown. We did not assess oxidative stress in our mutants as Lavatelli and colleagues did, but the gross developmental and physiological phenotypes are otherwise similar, if more severe, than the RNAi phenotypes (Lavatelli et al., 2020). Therefore, we conclude that *de novo* synthesis of LA by LIAS-1 is necessary for development and lifespan in *C. elegans* as it is in other organisms. The model generated here demonstrates the conservation of this pathway and presents a useful genetic model of lipoic acid deficiency.

## Methods

Animal maintenance and strain generation

Animals were maintained at 20°C on MYOB culture plates seeded with *E. coli* OP50 bacteria following standard practices ^3^ . *lias-1* deletion strains were generated via CRISPR/Cas9 gene editing as described in ^11^ . In brief, Cas9 protein, tracRNA, guide RNAs and a repair oligo were injected into the syncytial gonad of WT, day-1 adult hermaphrodites with a *dpy-10* co-CRISPR marker. Guide and repair sequences are listed in Table 1. Sterile *lias-1* alleles were balanced with the *qC1* balancer (see “Reagents”, below).

Genotyping analysis was completed with DNA lysates prepared via proteinase K lysis and amplified with NEB Taq Polymerase following the manufacturer's recommended protocol. Primer sequences were: 5' cacgtctgattgcgtatcgt 3' (forward primer) and 5' gacccatcgtttaccctgaaata 3' (reverse primer).

**Table 1: Oligos sequences for CRISPR. All reported 5' – 3'**

**Table:** 

**Oligo**	**Sequence (5'-3')**
*lias-1* 5' guide RNA	AGCATgcttaaacaatctacGUUUUAGAGCUAUGCUGUUUUG
*lias-1* 3' guide RNA	GTTCGCTCTTCGTACAAAGCGUUUUAGAGCUAUGCUGUUUUG
*lias-1* repair	gattttcaacacaaggagtttaattattaccggtaagccggagaattctatttgaaaaatgtgttgagaa
*dpy-10* guide RNA	gctaccataggcaccacgagGUUUUAGAGCUAUGCUGUUUUG
*dpy-10* repair	CACTTGAACTTCAATACGGCAAGATGAGAATGACTGGAAACCGTACCGCATGCGGTGCCTATGGTAGCGGAGCTTCACATGGCTTCAGACCAACAGCCTAT

Gene alignments

Genetic sequences were obtained from Eurofins Genomics. Sequence alignments were performed with T-Coffee and annotations were completed with JalView.

Imaging

Representative images of WT and mutant animals were captured on a Zeiss Stemi 508 equipped with a Zeiss Axiocam 208 color camera. Acquisition and image processing was completed with Zeiss Zen Blue 3.7 software on an HP Z2 desktop.

Lifespan and L3 arrest analyses

Synchronous populations for lifespan and L3 arrest assays were generated via synchronized egg lay. The day of the egg lay was considered day 0. Homozygous animals were manually picked to assay plates after hatching. For lifespan assays, fertile WT animals were transferred to fresh plates daily until the end of egg laying. All animals were transferred to fresh plates every other day thereafter. Lifespan assay with LA was completed identically to above with assay plates prepared as described below. For L3 arrest analyses, animals were assessed 72 hours after egg lay. Data from independent assays were aggregated into a complete data set.

Lipoic acid treatment

MYOB assay plates were supplemented with 0, 12.5, 25, 50, or 100 μM lipoic acid. Lipoic acid was prepared at 1,000X in 100% EtOH. All plates including the 0 μM plates contained 0.1% EtOH. Plates were otherwise treated as described above.

Western blot analysis

*C. elegans* and OP50 lysates were prepared with 100 µL RIPA buffer (ThermoFisher Scientific #J63306-AK) and 1X HALT protease and phosphatase inhibitor cocktail (ThermoFisher Scientific #78440). Samples were sonicated 5 times at 50% duty cycle with a tip sonicator and cellular debris was pelleted by centrifugation for 15' at 14,000 rpm at 4°C. Protein lysates were collected and frozen at -80℃ for storage. Protein concentration was quantified with a Pierce BCA Protein Assay Kit (ThermoFisher Scientific #002327). For each sample, 7.5 µg of protein was prepared in 1X Laemmeli Sample Buffer (Bio-Rad #1610747) and 5 mM DTT and run on polyacrylamide gels (Any kD Mini-PROTEAN TGX Stain-Free Gels, Bio Rad # 4569033) in Tris-Glycine-SDS buffer (Bio-Rad #1610732) prior to transfer to 0.2 mm nitrocellulose membranes (Bio-Rad # 1704158). Membranes were stained with 0.5% Ponceau S (w:v) in 5% glacial acetic acid for 5' at room temperature. Membranes were rinsed with distilled water to remove background and destained with 0.1% NaOH. Membranes were blocked with Everyblot Blocking Buffer (Bio-Rad #12010020) and incubated overnight at 4°C with the primary antibody (Rb anti-lipoic acid, 1:1,000, Calbiochem #437695-100 or Ms anti-alpha tubulin, 1:2,500, DSHB #12G10) diluted in 3% bovine serum albumin (BSA). Membranes were washed 5x5' with TBST before incubation with secondary antibody (HRP Goat anti-Rb or anti-Ms (1:15,000, Proteintech #SA00001-1 or SA00001-2, respectively) with StepTactin (1:50,000, Bio-Rad #1610381) in 3% BSA for 2 hours at room temperature. Antibodies were developed using Clarity Western ECL Substrate (Bio-Rad #1705060) with a Chemidoc MP.

Data analysis

Data were plotted and analyzed with GraphPad Prism version 10.3.1.

## Reagents

The strains used in this study are listed in Table 2. Strains are available upon request.

**Table 2: Strains used in this study**

**Table:** 

Strain	Genotype	Source
N2	WT	*Caenhorhabditis* Genetics Center
AG626	*lias-1 (av247)/ qC1 [ dpy-19 ( e1259 ) glp-1 ( q339 )] nIs189) III *	This study
AG627	*lias-1 (av248)/ qC1 [ dpy-19 ( e1259 ) glp-1 ( q339 )] nIs189) III *	This study
OP50	*Erischerichia coli* OP 50	*Caenhorhabditis* Genetics Center

## References

[R1] Ast Tslil, Meisel Joshua D., Patra Shachin, Wang Hong, Grange Robert M.H., Kim Sharon H., Calvo Sarah E., Orefice Lauren L., Nagashima Fumiaki, Ichinose Fumito, Zapol Warren M., Ruvkun Gary, Barondeau David P., Mootha Vamsi K. (2019). Hypoxia Rescues Frataxin Loss by Restoring Iron Sulfur Cluster Biogenesis. Cell.

[R2] Bick Nolan R., Dreishpoon Margaret B., Perry Ava, Rogachevskaya Anna, Bottomley Sylvia S., Fleming Mark D., Ducamp Sarah, Tsvetkov Peter (2024). Engineered bacterial lipoate protein ligase A (lplA) restores lipoylation in cell models of lipoylation deficiency. Journal of Biological Chemistry.

[R3] Brenner S (1974). The genetics of Caenorhabditis elegans.. Genetics.

[R4] Cao Xinyun, Hong Yaoqin, Zhu Lei, Hu Yuanyuan, Cronan John E. (2018). Development and retention of a primordial moonlighting pathway of protein modification in the absence of selection presents a puzzle. Proceedings of the National Academy of Sciences.

[R5] Cronan John E. (2016). Assembly of Lipoic Acid on Its Cognate Enzymes: an Extraordinary and Essential Biosynthetic Pathway. Microbiology and Molecular Biology Reviews.

[R6] Cronan John E. (2024). Lipoic acid attachment to proteins: stimulating new developments. Microbiology and Molecular Biology Reviews.

[R7] Cronan John E. (2020). Progress in the Enzymology of the Mitochondrial Diseases of Lipoic Acid Requiring Enzymes. Frontiers in Genetics.

[R8] Feng Dejiang, Witkowski Andrzej, Smith Stuart (2009). Down-regulation of Mitochondrial Acyl Carrier Protein in Mammalian Cells Compromises Protein Lipoylation and Respiratory Complex I and Results in Cell Death. Journal of Biological Chemistry.

[R9] Kastaniotis Alexander J., Autio Kaija J., R. Nair Remya (2020). Mitochondrial Fatty Acids and Neurodegenerative Disorders. The Neuroscientist.

[R10] Kropp Peter A., Wu Jing, Reidy Michael, Shrestha Sanjay, Rhodehouse Kyle, Rogers Philippa, Sack Michael N., Golden Andy (2021). Allele-specific mitochondrial stress induced by Multiple Mitochondrial Dysfunctions Syndrome 1 pathogenic mutations modeled in Caenorhabditis elegans. PLOS Genetics.

[R11] Lavatelli Antonela, de Mendoza Diego, Mansilla María Cecilia (2020). Defining Caenorhabditis elegans as a model system to investigate lipoic acid metabolism. Journal of Biological Chemistry.

[R12] Mayr Johannes A., Feichtinger René G., Tort Frederic, Ribes Antonia, Sperl Wolfgang (2014). Lipoic acid biosynthesis defects. Journal of Inherited Metabolic Disease.

[R13] Mayr Johannes A., Zimmermann Franz A., Fauth Christine, Bergheim Christa, Meierhofer David, Radmayr Doris, Zschocke Johannes, Koch Johannes, Sperl Wolfgang (2011). Lipoic Acid Synthetase Deficiency Causes Neonatal-Onset Epilepsy, Defective Mitochondrial Energy Metabolism, and Glycine Elevation. The American Journal of Human Genetics.

[R14] McCarthy Erin L., Booker Squire J. (2017). Destruction and reformation of an iron-sulfur cluster during catalysis by lipoyl synthase. Science.

[R15] McCarthy Erin L., Rankin Ananda N., Dill Zerick R., Booker Squire J. (2019). The A-type domain in Escherichia coli NfuA is required for regenerating the auxiliary [4Fe–4S] cluster in Escherichia coli lipoyl synthase. Journal of Biological Chemistry.

[R16] Pradel Laura S., Ho Yu-Lin, Gohlke Holger, Kassack Matthias U. (2024). The Antioxidant and HDAC-Inhibitor α-Lipoic Acid Is Synergistic with Exemestane in Estrogen Receptor-Positive Breast Cancer Cells. International Journal of Molecular Sciences.

[R17] Tort Frederic, Ferrer‐Cortes Xènia, Ribes Antonia (2016). Differential diagnosis of lipoic acid synthesis defects. Journal of Inherited Metabolic Disease.

[R18] Warnhoff Kurt, Hercher Thomas W., Mendel Ralf R., Ruvkun Gary (2021). Protein-bound molybdenum cofactor is bioavailable and rescues molybdenum cofactor-deficient
*C. elegans*. Genes & Development.

[R19] Warnhoff Kurt, Ruvkun Gary (2019). Molybdenum cofactor transfer from bacteria to nematode mediates sulfite detoxification. Nature Chemical Biology.

[R20] Warui Douglas M., Sil Debangsu, Lee Kyung-Hoon, Neti Syam Sundar, Esakova Olga A., Knox Hayley L., Krebs Carsten, Booker Squire J. (2022). In Vitro Demonstration of Human Lipoyl Synthase Catalytic Activity in the Presence of NFU1. ACS Bio & Med Chem Au.

